# Synthesis and characterization of an electrochromic copolymer based on 9, 10-di (furan-2-yl)anthracene and 3,4-ethylenedioxythiophene

**DOI:** 10.55730/1300-0527.3419

**Published:** 2022-03-19

**Authors:** Ali KALÇIK, Arif KIVRAK, Buket BEZGİN ÇARBAŞ

**Affiliations:** 1 Department of Energy Systems Engineering, Karamanoğlu Mehmetbey University, Karaman, Turkey; 2 Conductive Polymers and Energy Applications Laboratory, Karamanoğlu Mehmetbey University, Karaman, Turkey; 3 Department of Chemistry, Faculty of Sciences, Eskişehir Osmangazi University, Eskişehir, Turkey

**Keywords:** Conducting polymers, electrochemical copolymerization, electrochromism, electrochromic materials, anthracene

## Abstract

In this study, a new copolymer, which is a combination of two chemical structure (9,10-di(furan-2-yl)anthracene and 3,4-ethylenedioxythiophene was synthesized via electrochemical synthesis methods in the electrolyte medium of 0.1 M TBAPF_6_/ACN solution. PEDOT homopolymer was also electrosynthesized for comparator experiments of the polymer formations in the same medium. The characterizations of polymers were achieved with general optical and electrochemical characterization techniques. The corresponding electrochromic copolymer shows blue and lilac color in its neutral and oxidized states, respectively. The copolymer has an optical band gap of 1.65 eV and 24% optical contrast at 500 nm with a high coloration efficiency (170 cm^2^ /C) and a fast switching time (1.0 s).

## 1. Introduction

Nowadays, studies on plastic polymers have shown that there are many application areas of this kind of materials, where they are used due to their high chemical, mechanical and thermal properties as well as being cheap and portable. After it became evident that the polymers could be conductive, the range of applications of the polymers was further expanded and becomes a breakthrough in solving the technological challenge and health problems of humanity. Conductive polymers, which sometimes compete with superconducting materials, are used in many application fields. The most popular of these are sensors [1–4], solar cells [5], light emitting devices [6], electrochromic devices [7–10], capacitors [11]. Among them, electrochromic property of conjugated polymers was built into energy storage applications, such as solar cells, sensors and supercapacitors [12–14]. Moreover, hybrid applications have been very popular in terms of energy efficiency which is considered as one of the renewable energy sources. Therefore, many scientists have tried to find and synthesize new conjugated polymers in order to use them in these kinds of applications. This is the basis for the production of new synthesis approaches. While the first studies were homopolymerization of conjugated monomers, the subsequent studies were related with the combination of many properties in one material as well as to make polymerization easily. For that reason, the usage of copolymerization technique in order to achieve the desired properties (band gap, stability, color chart, fast switching, etc.) opened a new door for scientists in the desired application areas of conductive polymers. Meanwhile, two famous approaches have been used for copolymerization. The first approach is to copolymerize the comonomer with the D-A-D method and to obtain copolymer chains having regular monomer sequences. The other approach is to obtain different monomer sequential copolymers by taking two conjugated monomers (one of this is electron-rich heterocycles such as thiophene (Th), 3,4-ethylenedioxythiophene (EDOT) and 3,4-propylenedioxythiophene (ProDOT)) with different concentration ratios, which they have neighboring oxidation potentials for polymerization. There are many example studies related with this approach in literature [7, 8, 15–18].

Poly aromatic, anthracene has been focused attention because of its photoluminescence and electroluminescent properties [19, 20]. There are many studies related with variety of anthracene-based organic compounds, which compose of different active side groups [21, 22]. When thiophene, furan, 3,4-ethylene dioxythiophene (EDOT) and 3,4-propylenedioxythiophene (ProDOT) groups were added into 9 and 10 positions of anthracene molecules, the degree of conjugation increase and the oxidation potential of the structure decreases [23–27]. Among them, polymers with EDOT chemical structure stand out with features such as lower half wave potential, electrochemical and thermal stability upon cycling. Moreover, electrochromic PEDOT derivatives possess fast switching time with high optical contrast [28]. However, there are a few studies about the applications of furanyl anthracene based organic molecules [29–31]. Recently, we reported that furan-anthracene-furan (DFA) acceptor type structures could be novel small organic molecules for organic solar cell applications [32].

Herein, a new copolymer of furan-anthracene-furan structures was designed and synthesized. Firstly, furan-anthracene-furan was tried to be electropolymerized, but we did not achieve. Then, furan-anthracene-furan (DFA) was copolymerized with EDOT unit. It is expected that the combination of two chemical structures in a polymer chain will enhance the polymer chain backbone with superior electrochemical and spectroelectrochemical performance properties of PEDOT polymer film.

## 2. Materials and methods

All chemicals were used from Sigma Aldrich without further purification, (9,10-di(furan-2-yl)anthracene comonomer was synthesized according to reference [25, 28]. In order to copolymerize (9,10-di(furan-2-yl)anthracene with 3,4-ethylenedioxythiophene (EDOT), 10 mM concentration was used for each chemicals. Acetonitrile (ACN) and 0.1 M tetrabutylammonium hexafluorophosphate (TBAPF_6_) were used as solvent and electrolyte during electrocopolymerization and optical and electrochemical characterization, respectively. Platinum disc (0.02 cm^2^) as working electrode, platinum wire as counter electrode and Ag/AgCl reference electrode were used during electrochemical analysis. In the case of spectroelectrochemical analysis, an indium-tin oxide (ITO, Delta, Tech. 8–12 Ω, 0.7 cm × 5 cm) as working electrode, Pt wire as counter electrode and Ag wire as reference electrode were used. A Teflon cape, which covers three holes on, were placed in a quartz UV-cuvette. As a potentiostat, an Ivium CompactStat brand device was used during electrochemical and spectroelectrochemical studies. For spectroelectrochemical studies, a Specord S600 spectrometer was used. The color space given by the International Commission of Illumination with luminance (L), hue (a), and intensity (b) was also analyzed with this brand device. FTIR spectra were carried out with Bruker Equinox 55 with an attenuated total reflectance (ATR).

## 3. Results

### 3.1. Electrochemical polymer and copolymer synthesis

**Figure 1:**
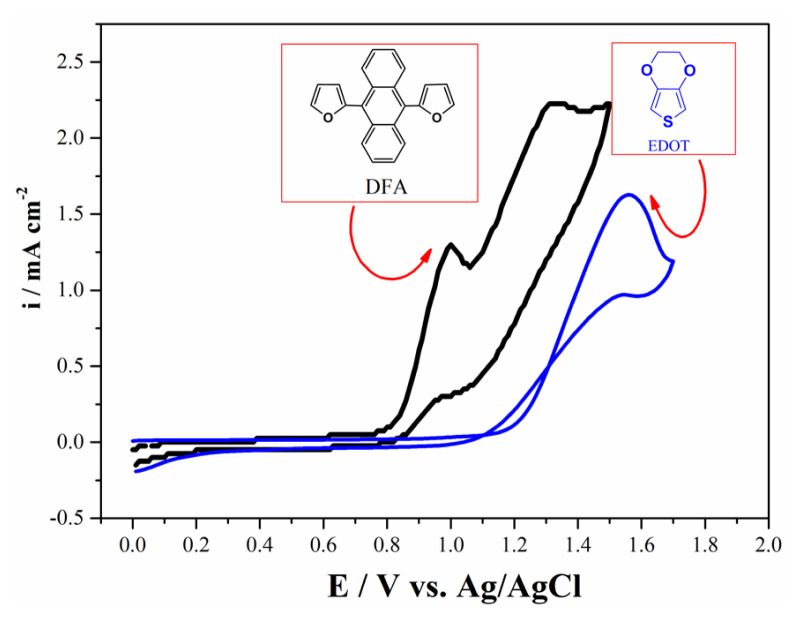
Cyclic voltammogram of DFA (10 mM) on Pt disc electrode in the medium of 0.1 M TBAPF_6_/ACN solution with a scan rate of 100 mV s^−1^ vs. Ag/AgCl.

**Figure 2:**
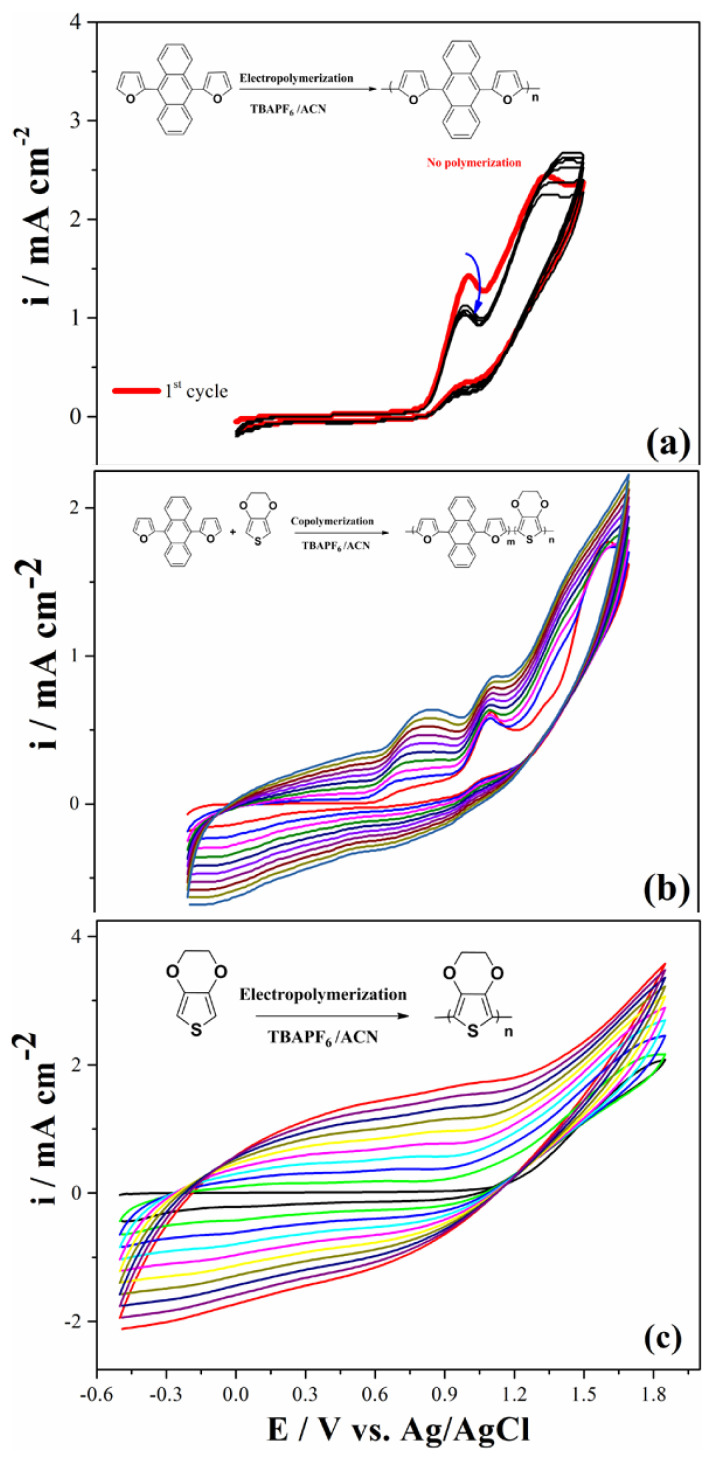
Electropolymerization of (a) DFA and EDOT monomers with a monomer feed ratio (DFA:EDOT = 3:2) (b) EDOT solution with a scan rate of 100 mV s^−1^ vs. Ag/AgCl in 0.1 M TBAPF_6_/ACN solution.

Initially, the electrochemical behavior of DFA was carried out in the medium of 0.1 M TBAPF_6_/ACN at a scan rate of 100 mV s^−1^ vs. Ag/AgCl via cyclic voltammetry method (CV). DFA monomer was cycled in the positive potential region and two quasi-reversible oxidation potentials at 1.0 V and 1.32 V were observed, respectively (Figure 1). DFA monomer was tried to be electropolymerized via general electroanalytical techniques. However, any method or medium was enough to be electropolymerized (Figure 2a). For that reason, copolymerization technique was used in order to add DFA unit into polymer chain backbone. A neighbor oxidation potential of two monomers is an important point in order to get the electrocopolymerization process [33]. The oxidation potential of EDOT was 1.55 V vs. Ag/AgCl using the same CV conditions with those of DFA (Figure 1).

A small oxidation potential difference (0.23 V) between two monomers is acceptable in order to get copolymerization. Furthermore, the electrochemical and spectroelectrochemical properties of corresponding polymer were compared with those of PEDOT polymer for comparison reasons. A comonomer mixture with a feed ratio of DFA/EDOT (3:2) was prepared in the medium of 0.1 M TBAPF_6_/ACN. The different copolymerization conditions were based on the optimization study for feed ratio of monomers undertaken and this condition (a feed ratio of DFA/EDOT (3:2)) provided the appropriate rate for copolymerization. In the first cycle, three oxidation potentials (1.0 V, 1.35 V, and 1.53 V) for mixture were observed. After ten successive scans for this comonomer mixture in medium of 0.1 M TBAPF_6_/ACN with a scan rate of 100 mV s^−1^, the current intensities of peaks increase for each next cycle showing that an electroactive polymer were coated on the working electrode (Figure 2b). PEDOT polymer also synthesized electrochemically in a potential range of −0.5 V and 1.8 V vs. Ag/AgCl.

**Figure 3:**
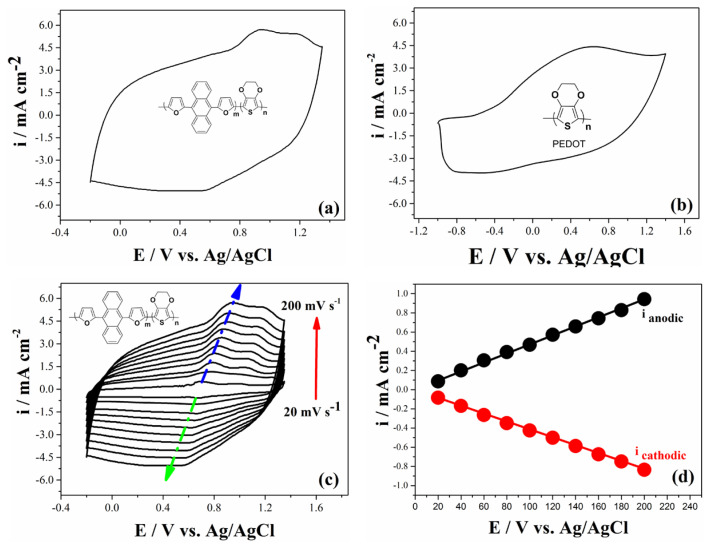
Electrochemical characterization behavior of (a) copolymer and (b) PEDOT on Pt disc electrode in the monomer free medium at a scan rate of 200 mV s^−1^. (c) CV data of copolymer with different scan rates between 20 mV s^−1^ and 200 mV s^−1^ with an increment of 20 mV s^−1^ PEDOT in the monomer free medium. (d) Relationship of anodic (i_anodic_) and cathodic (i_cathodic_) current peaks as a function of scan rate.

PEDOT and furan anthracene based copolymer coated on the working electrodes were cleaned in a monomer free solvent medium in order to get rid of monomers, which were not polymerized. The electrochemical behaviors of copolymer and PEDOT polymers were recorded in monomer free medium (0.1 M TBAPF_6_/ACN) via CV method with a scan rate of 200 mV s^−1^. Copolymer shows one reversible redox couple presenting the doping (0.90 V) and dedoping (0.40 V) while PEDOT polymer shows different potentials at 0.60 V and −0.60 V for doping and dedoping processes, respectively (Figures 3a and 3b). Since the copolymer chains possess more EDOT and DFA monomers in its chains, the charge density of copolymer is higher than that of PEDOT.

The kinetic behavior of polymer films can be analyzed from CV experiments of the polymer films. Figure 3c shows the electropolymerized copolymer behavior in different scan rates between 20 mV s^−1^ and 200 mV s^−1^ with an increment of 20 mV s^−1^. It is found that the current densities of both doping and dedoping processes ascend linearly with increasing scan rate, indicating that a copolymer film on working electrode were well coated and the redox behavior is a nondiffusional process (Figure 3d).

### 3.2. Electrochromic properties of copolymer

**Figure 4:**
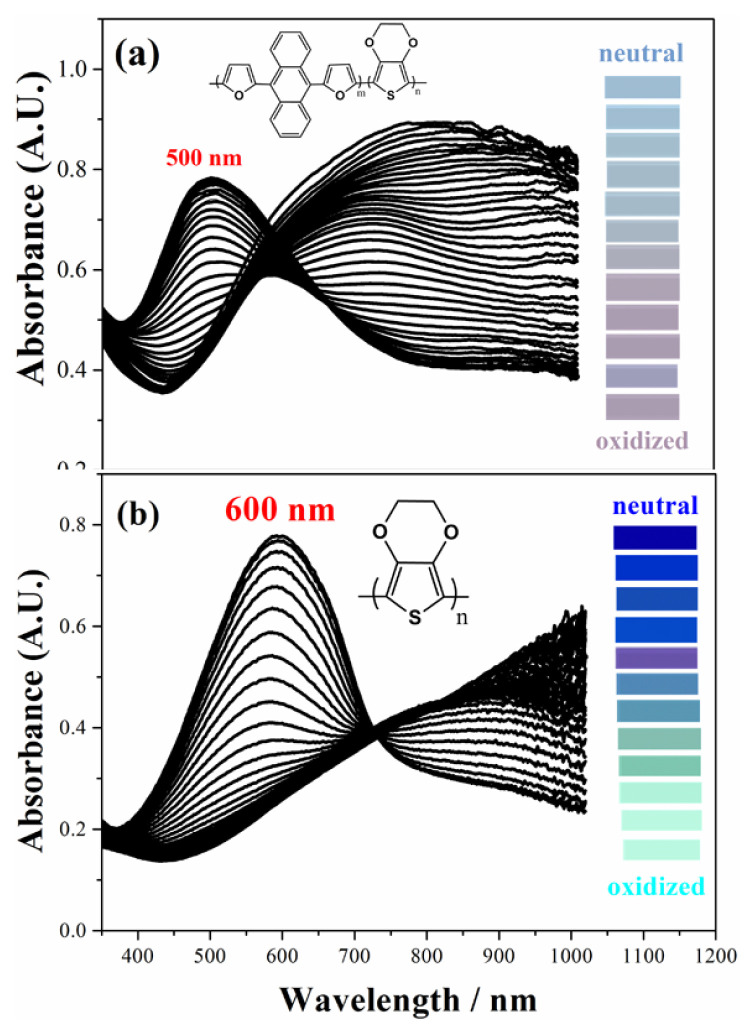
Spectroelectrochemical studies of (a) copolymer (b) PEDOT and inset figures; the colors of the copolymer and PEDOT polymer films at their neutral and oxidized states in 0.1 M TBAPF_6_/ACN solution.

Spectroelectrochemical behavior study, which is a concurrent execution of UV-vis spectroscopy with potentiostat kinetically or not, was detected in order to get information about band gap, interband charge states of conductive polymers that form during doping and dedoping for electrochromic behavior. For this purpose, copolymer and PEDOT polymer (ten cycled) were electrosynthesized on ITO working electrode. The polymer coated on ITO working electrode, Pt and Ag wires as counter and reference electrode, respectively, were placed on Teflon cover with three holes for electrodes entrances and Teflon cape with three electrodes set up were inserted into a quartz cell filled with 0.1 M TBAPF_6_/ACN electrolyte solvent medium. The absorption spectrum was monitored during potential cyclic scan for both copolymer (Figure 4a) and PEDOT polymer (Figure 4b). Furthermore, copolymer and PEDOT display bands at 500 nm and 600 nm, respectively in their neutral states, indicating π-π* transitions. UV-vis spectrum for the PEDOT polymer in the π-π* transition showed a hypsochromic shift (from 600 nm to 500 nm) when compared with UV-vis spectrum for the copolymer. Upon electropolymerization, the absorption band of copolymer shift to a longer wavelength due to time required to the intramolecular charge transfer between units in the copolymer chain. An increase of DFA content in polymer chain and steric effect of bulky groups, like anthracene in DFA comonomer structure, causes low degree of polymerization. The band gap value of copolymer (1.65 eV) is a little bit higher than that of PEDOT polymer (1.62 eV) found as from the commencement on the low energy band end and lower or higher than that of its analogs in Table. After doping of neutral conjugated polymers, while copolymer shows a simultaneous increase at about 750 nm (polaron formation) and upon further doping, a new band at about 800 nm (bipolaron formation) appears, PEDOT also has polaron and bipolaron charge carriers at about 900 nm and 1050 nm, respectively. As seen in Figures 4a and 4b, the copolymer has completely different spectroelectrochemical behavior when compared to that of PEDOT polymer because of copolymerization.

Electrochromic properties of polymers can be specified with regard to CIE 1976 (L, a, b) color space with daylight (Standard Illuminant D65/10 as illuminant and 10°). L, a and b are interrelated with the parameter of the lightness, red-green and yellow-blue balances, respectively [34]. While the colors copolymer in the neutral and oxidized states are blue (L = 64.52; a = 8.52; b = −8.82) and lilacs (L = 73.57; a = −6.63; b = −13.11), respectively, PEDOT polymer has color transition from dark blue (L = 17.88; a = 48.08; b = 79.32) to transparent blue (L = 90.56; a = −22.05; b = 1.29) (oxidized). Based on the color data, the colors of the PEDOT polymer chain in different redox states change as DFA unit entrance occurs. One hundred nanometers blue shift of copolymer with respect to that of PEDOT in π-π* transition at neutral state is the reason for color change of copolymer.

**Figure 5:**
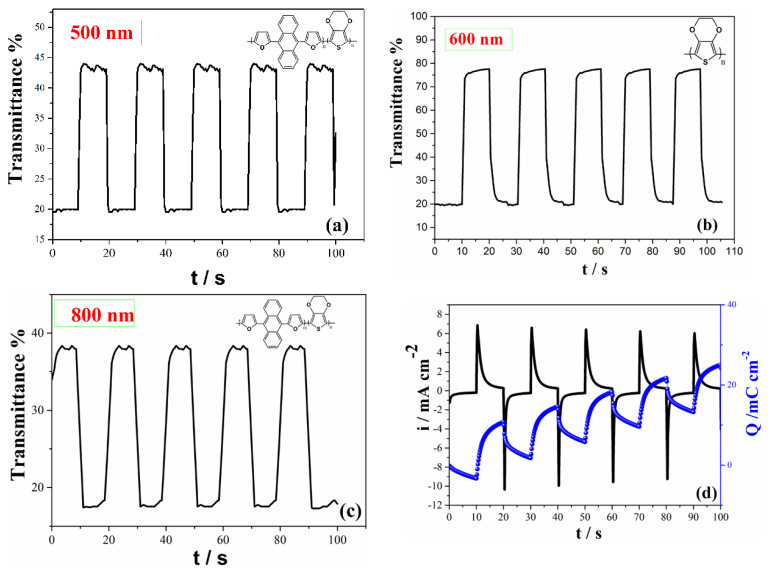
Optical contrast study experiments for copolymer at a) 500 nm c) 800 nm, for PEDOT polymer at b) 600 nm d) charge density and current density of copolymer switched for 10 s between their neutral and oxidized states.

Electrochromic applications can be characterized with some parameters such as coloration efficiency (CE), switching time (t_switching_) and optical contrast (ΔT%). The data related with colors switching between oxidized and neutral states for polymer via square wave potential method at their maximum absorbance wavelength data were given in Table. Both copolymer and PEDOT was switched at the potential ranges between −0.20 V and 1.30 V for 10 s at 500 and 800 nm (Figures 5a and 5c). The switching charge and current data vs. time was also given in Figure 5d for copolymer. A similar technique for PEDOT was employed at 600 nm and switched between its neutral and oxidized states (Figure 5c). These results show that this copolymer is very amenable for the electrochromic applications.

In Table, there are some spectroelectrochemical properties of anthracene based copolymer formations with EDOT derivatives. Anthracene derivatives with different donor units copolymerized with EDOT unit copolymers were systematically described and compared with our new synthesized copolymer structure. Firstly, anthracene and EDOT copolymer properties were examined. The changes are discussed with the different donor groups such as thiophene, EDOT, ProDOT and furan. As seen in Table, the band gap of polymers decrease as the donor unit changes. The maximum absorption wavelength (λ_max_) value for polymers also change due to the chemical structure of polymers as EDOT structure entrance into the polymer chain and λ_max_ shifts to the higher λ_max_ values since that of PEDOT polymer has a λ_max_ of 600 nm. While the percent transmittance (%ΔT) of DFA based copolymer was found as 24 at 500 nm and 20 at 800 nm, the switching time of the copolymer has a little bit lower switching time (1.0 s at 500 nm and 2.2 s at 800 nm), compared with other derivatives (the data given the time needed to reach 95% of the total transmittance change for polymers). Because each copolymer structure has different capability of electroactivity and characteristic morphology, easy entrance or extraction ability of the dopant ions into copolymer structure change. This affects switching speed of the copolymer films [35]. Moreover, higher coloration efficiency values than that of others (170 cm^2^/C at 500 nm and 102 cm^2^ /C at 800 nm ) were found (Table).

### 3.3. Chemical structure analysis of copolymer

**Figure 6:**
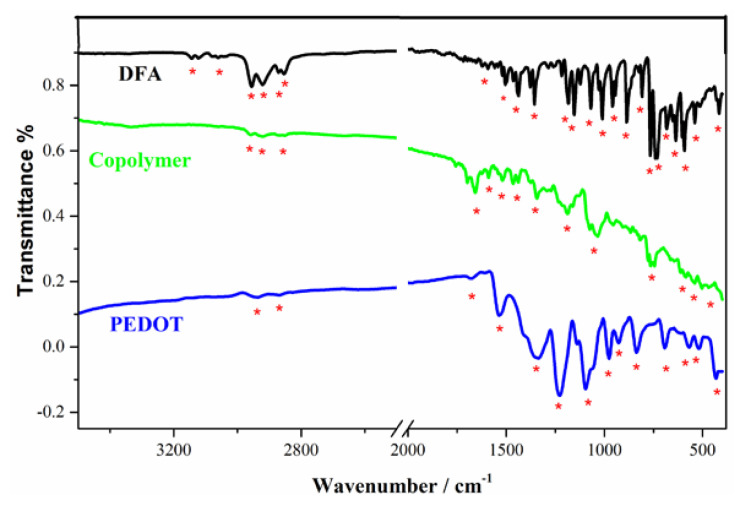
FTIR spectrum of DFA, copolymer and PEDOT polymers.

In Figure 6, DFA has following absorption peaks related with stretching modes of C-H aromatic rings and C-O of furan units: 3143, 3052, 2954, 2911, 2848, 1602, 1504, 1445, 1352, 1190, 1151, 946, 877, 809, 765, 735, 686, 637, 579, 535, 422 cm^−1^. The FTIR spectrum of copolymer and PEDOT were also added into Figure 6 for comparison reasons. The synthesized copolymer has the characteristic peaks in the wavenumber range 1250 cm^−1^ and 1500 cm^−1^ with that of DFA, which indicates that DFA (furan-anthracene-furan) structure enters into copolymer chain. Both polymer spectrum show broad bands as many conjugated polymers have. The FTIR spectrum of copolymer like that of PEDOT has a stretching vibration band at about 1670 cm^−1^ corresponds to polyconjugation, which is an evidence of copolymerization of DFA and EDOT.

## 4. Conclusion

In the present study, a new copolymer based on furanyl anthracene and EDOT units was electrochemically synthesized. The incorporation of EDOT into polymer chain backbone was also proved with electroanalytical, spectroelectrochemical and chemical structural method by comparing those of PEDOT polymer. Band gap of copolymer was found as 1.65 eV. It was found that electrochromic copolymer had a light blue color in the fully reduced form and a lilac color in the fully oxidized state with a coloration efficiency of 170 cm^2^/C at 500 nm and 102 cm^2^/C at 800 nm in a low switching time (1.0 s at 500 nm and 2.2 s at 800 nm). All of these indicate that the copolymers have been proposed for use in electrochromic devices.

## Figures and Tables

**Table:** Spectroelectrochemical properties of copolymer and other similar chemical structures.

Polymer [ref]	λ_max_ (nm)	% ΔT	t_switching_ (s)	E_g_^opt^ (eV)	CE (cm^2^/C)	Colors	
						Neutral	Oxidized
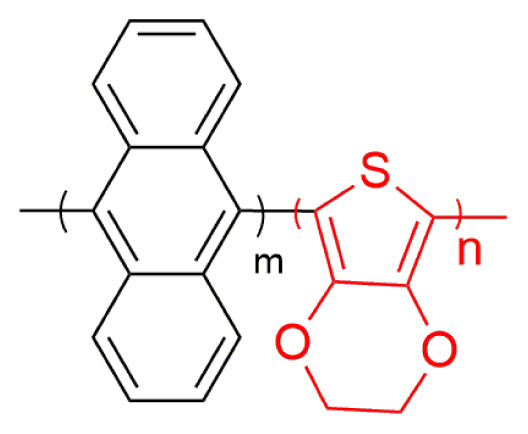 [25]	470 507	73 (507 nm)	2.3 (507 nm)	1.77 *	278 (507 nm)	Red	Dark blue
			2.1 (797 nm)		198 (780 nm)		
			4.7 (942 nm)		286 (942 nm)		
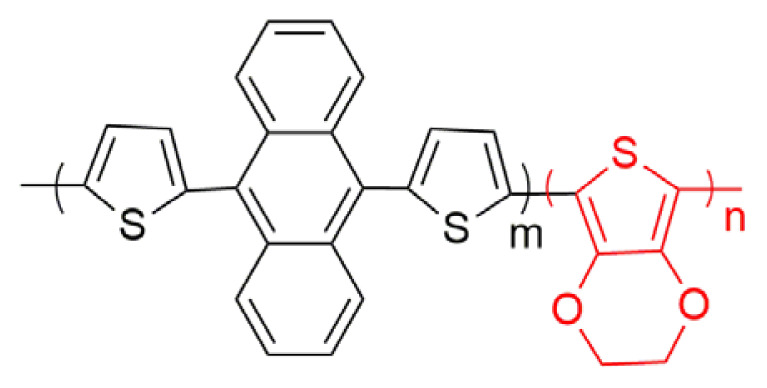 [24]	535	35.6 (535 nm)	1.9 (535 nm)	1.73	-	Purple	Blue
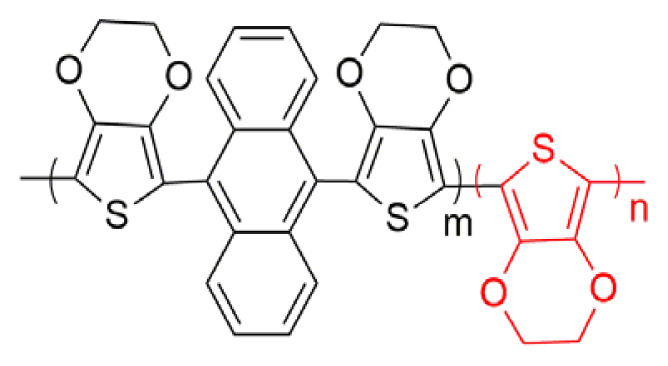 [27]	519** 544 ** 588 **	11	2.1	1.579 ** 1.584 ** 1.590 **	-	Red	Blue
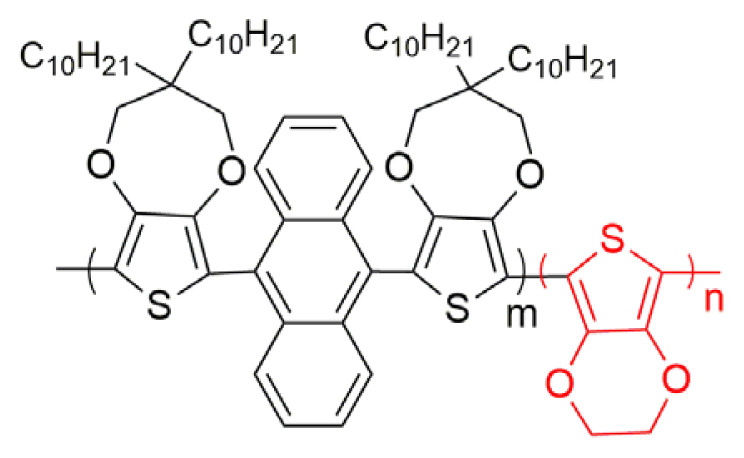 [26]	580	49.0 (580 nm)	0.7 (580 nm)	1.65	142 (580 nm)	Navy blue	Dark cyan
		63.0 (1000 nm)					
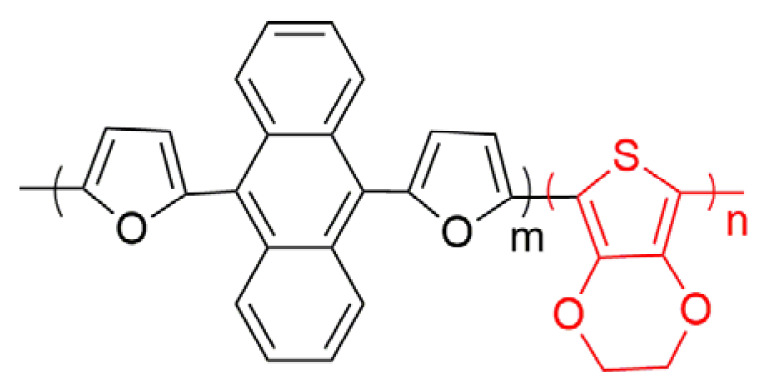 [This work]	500	24.0 (500 nm)	1.0 (500 nm)	1.65	170 (500 nm)	Light blue	Lilac
		20.0 (800 nm)	2.2 (800 nm)		102 (800 nm)		

* Band gap data was predicted by using spectroelectrochemical study graph at reference [25].

** The copolymer formation were synthesized with different three oxidation potentials at reference [24].

For that reason, there are three λ_max_ and their E_g_^opt^ values.
